# “Surviving against the odds. The impact of peer support workers on a chronically suicidal adolescent in secure residential youth care: a single case report from the Netherlands”

**DOI:** 10.1080/17482631.2024.2409514

**Published:** 2024-10-08

**Authors:** Shireen P. T. Kaijadoe, Karin S. Nijhof, Helen Klip, Arne Popma, Ron H. J. Scholte

**Affiliations:** a Karakter Child and Adolescent Psychiatry University Centre, Nijmegen, the Netherlands; b Behavioural Science Institute, Radboud University Nijmegen, Nijmegen, the Netherlands; c Research Department, Pluryn, Nijmegen, the Netherlands; d Child and Adolescent Psychiatry & Psychosocial Care, Amsterdam UMC, Vrije Universiteit Amsterdam, Amsterdam, the Netherlands

**Keywords:** Suicidal adolescents, secure residential youth care, peer support workers, qualitative single case design, recovery oriented intervention

## Abstract

**Background:**

The use of peer support workers to support suicidal adolescents is underdeveloped. This study focuses on the effects of a one-year intervention with peer support workers on a chronically suicidal adolescent residing in a secure residential youth care facility in the Netherlands. Moreover, we explore the mechanisms that underpin the role of peer support workers in detail.

**Method:**

This study employed a single case study design. We conducted seven semi-structured interviews with staff, peer support workers, and a chronically suicidal adolescent. The interviews were analysed using a thematic analysis.

**Results:**

The results indicate that the suicidal tendencies of the adolescent decreased significantly one year after the peer support intervention compared to the initial baseline. Working mechanisms that underpinned the peer support intervention emphasized building meaningful and trust-based relationships, providing recognition and hope, and practical support from a recovery-oriented perspective.

**Conclusion:**

The results suggest that peer support has a beneficial impact on the adolescent and treatment teams. Peer support workers contribute to a sense of belonging and connection, coping with suicidality, rediscovering life goals, and improving adolescent self-management. Barriers and facilitators to implementing peer support workers are also discussed.

## Introduction

Suicide is a major public health concern worldwide, with over 700,000 fatalities occurring annually (World Health Organization, 2021). Research shows that a sharp increase in the number of suicide deaths throughout the lifespan occurs between early adolescence and young adulthood (Cha et al., 2018; Nock et al., 2008). Moreover, the increased psychosocial stressors during the COVID-19 pandemic have heightened the vulnerability of adolescents to stressful situations, leading to an elevated risk of suicidal behaviour in this population (Ambrosetti et al., 2021; Amerio et al., 2020). In the Netherlands, suicide is the main cause of death among young people aged 10–20 years (Central Bureau of Statistics, 2020). Young people with suicidal behaviours can be referred to Secure Residential Youth Care (SRYC) facilities (Buysse et al., 2019). Admission to SRYC typically occurs when a young person is deemed to be at risk of harm to themselves or others and when the person requires a more secure environment than a traditional residential care setting. The aim of SRYC is to guarantee adolescent safety and prepare residents to return to and participate in society (Whittaker et al., 2016).

Approximately one-third of adolescents in residential youth care exhibit behaviours such as self-harm, talking about or expressing thoughts of suicide, and previous suicide attempts (Duppong Hurley et al., 2014; Vermaes et al., 2014), SRYC facilities have implemented suicide prevention protocols and risk assessment programmes to respond to suicidal behaviours and crises. SRYC treatment encompasses therapeutic activities within a living group. SRYC institutions add more specialized services, such as individual therapies including trauma therapy, counselling, and on-site pharmacotherapy when indicated (Whittaker et al., 2015). Teams are typically multidisciplinary and led by psychologists (Ministerie van Volksgezondheid Welzijn en Sport, 2019).

Notwithstanding a decrease in the use of coercive measures (van Dorp et al., 2021), professionals in SRYC still draw on such measures as a last resort in case of incidents, such as suicidal threats, self-harm, aggressive behaviour, or rule violation for safety reasons (Whittaker et al., 2015). Restriction measures typically involve physically restraining an individual, temporary isolation, examination of the body or clothing, urine control, or checking residents’ rooms for prohibited objects. Coercive measures such as involuntary seclusion have a negative impact on suicidal adolescents, as they lead to withdrawal, distrust, and non-disclosure, exacerbating suicidal feelings and endangering the therapeutic relationship (Fisher, 1994; Haugom et al., 2019; Kaijadoe et al., 2023; LeBel et al., 2010). Moreover, it also increases the risk of exacerbating traumatic symptoms and hampering the fulfilment of important psychological needs such as connectedness and autonomy (Kaijadoe et al., 2023). Although efforts have been made to reduce and prevent seclusion, it remains a commonly used practice in many SRYC organizations (van Dorp et al., 2021). Hence, these measures have far-reaching implications, necessitating a mandatory evaluation by the juvenile judge to determine the appropriateness of enforced admission into SRYC (Dresen et al., 2017).

In the Netherlands, the Child and Youth Act [Jeugdwet], enacted in 2015, governs secure residential youth care. This legislation aims to achieve several key objectives: (1) enhance the problem-solving abilities of children, young individuals, parents, and their social circles; (2) foster parental capabilities and support within the social environment; (3) emphasize prevention and early detection; (4) deliver timely and tailored assistance; and (5) promote effective and efficient collaboration with families. Despite the availability of therapeutic services in SRYC, there is limited empirical evidence demonstrating the effectiveness of residential care programmes in achieving positive treatment outcomes (Harder & Knorth, 2015; Leipoldt et al., 2019). While several meta-analyses have shown the modest effectiveness of SRYC in addressing youth behavioural issues (de Swart et al., 2012; Strijbosch et al., 2014), critics have recently expressed concerns about its suitability for achieving the aforementioned goals (Whittaker et al., 2016). Recent research conducted by Gutterswijk et al. (2023) revealed that the majority of adolescents referred for SRYC treatment did not experience significant benefits (Gutterswijk et al., 2023). Furthermore, residents report that treatment programmes often fail to meet their individual needs (Brimblecombe et al., 2015; Kaijadoe et al., 2023; Nolbeck et al., 2020a, Royal College of Psychiatrists, 2010). Additionally, the effectiveness of youth care seems to have reached a plateau, with limited improvement in effect size (Jones et al., 2019). Therefore, there is an urgent need to develop more effective treatment strategies. One potential option is the utilization of peer support workers (Mead & Filson, 2017). Peer support workers are clients or former clients who are trained and educated to transform their personal, lived experience as a client into “experiential knowledge” that helps other clients (Leemeijer & Noordegraaf, 2020).

The use of peer support workers is becoming increasingly common in child and adolescent mental health services (Gopalan et al., 2017; Lenkens et al., 2020; Mulfinger et al., 2018). The general idea of peer support is based on the belief that people who have faced, endured, and overcome adversity can offer useful support, encouragement, and hope to others in similar situations. The employment of peer support workers within mental health organizations is becoming increasingly common, both internationally (Gillard et al., 2013) and nationally (Baillergeau & Duyvendak, 2016). The scoping review by de Beer et al. (2022) provides an excellent overview of the growing number of studies on peer support workers in treatment settings (de Beer et al., 2022), but it did not include literature on peers supporting suicidal adolescents in secure residential youth care (SRYC). This lack of research is notable given the high prevalence of suicidal behaviour among SRYC residents and the fact that suicidal adolescents in SRYC stress the need to talk to peer support workers to overcome suicidality (Kaijadoe et al., 2021).

Scientific journals regularly highlight the importance of deploying peer support workers for suicide prevention and the need for further scientific research in this area (Salvatore, 2010; Thomas, 2011). Theoretically, peer support workers who have lived experiences with suicidality could make an important contribution to suicide prevention (Mead et al., 2001; Salvatore, 2010; Thomas, 2011). Peer support workers offer the opportunity to find and create new meaning for individuals facing suicidal tendencies through trusted relationships and conversations that lead to new ways of understanding crises (Mead & Filson, 2017). For example, experts with lived experience could contribute to breaking the stigma surrounding suicidality and provide unique perspectives on support and empowerment (Salvatore, 2010). Peer support workers can also contribute to self-management, a sense of belonging and connection, coping with suicidality, and the rediscovery of life goals (Chi et al., 2014; Davidson et al., 2012). Research of Niederkrotenthaler et al. (2022) provided convincing evidence that narratives of hope and recovery from suicidal crises have beneficial effects on suicidal ideation (Niederkrotenthaler et al., 2022).

However, research examining the processes through which peer support benefits are achieved is underdeveloped (Watson, 2019). Therefore, we aimed to explore in detail the mechanisms underlying the role of peer support workers. We used a single case design as it delves deep into details, that might be overlooked in broader studies (see study design) (Zuidersma et al., 2020). This study combines the reflections, observations, and knowledge gathered from semi-structured interviews with staff, peer support workers, and a service user. The objective of this paper is to describe the impact of peer support work on the suicidal behaviour of a chronically suicidal adolescent residing in an SRYC facility in the Netherlands. Additionally, we explored the barriers and facilitators of implementing peer support workers in the practice of secure residential youth care. Finally, we hope to offer guidance and practical advice to other SRYC-services that consider employing peer support workers to enhance outcomes for adolescents with chronic suicidality residing in SRYC in the Netherlands.

Suicide is defined as the act of an individual intentionally ending their own life, and we use the term suicidal behaviour to refer to thoughts and behaviours related to intentionally taking one’s own life (O’Connor & Nock, 2014). The surnames used in this study are fictive.

## Method

### Study design

This qualitative study employed a phenomenological, single case design (Creswell, 2018), to explore the experiences of a suicidal adolescent, two peer support workers, and four staff members in a Dutch Secure Residential Youth Care (SRYC) facility, all of whom were involved in the peer support worker intervention. Prior to this study, there was a lack of available data regarding the impact of peer support workers supporting chronically suicidal adolescents in SRYC. Single case designs can evaluate experimental interventions for individual clients (Kazdin, 1978). While case reports are considered a lower level of evidence in scientific literature, they provide valuable insights that can enhance patient care (Alsaywid & Abdulhaq, 2019). However, evaluating treatment effects in case reports is challenging due to limited control over threats to internal validity (Heyvaert et al., 2017). Subjective evaluation methods were used to evaluate the extent of behavioural changes achieved during and after the intervention (Kazdin, 1978).

### Research setting

This study was conducted within an SRYC organization in the Netherlands. VISTOS stands for “Very Intensive Short-Term Observation and Stabilization unit” (in Dutch “ZIKOS”). This unit caters to young individuals who require SRYC and who experience severe psychiatric distress. The primary objective of this unit is to observe and stabilize individuals. The key distinction between this unit and the standard SRYC group lies in the extended periods of planned isolation (room placement) experienced by young people with suicidal tendencies in the VISTOS ward. The target demographics for VISTOS primarily comprise nearly 100% of suicidal adolescents. In the specific case study, the adolescent under examination had resided in the VISTOS ward for 15 months.

### Case description: rose

Rose (fictive name) was 15 years old when she was involuntarily admitted to SRYC. Rose has a developmental history of insecure attachment, early trauma, and anxiety, resulting in significant psychological damage and suicidality. After multiple severe suicide attempts, she was placed in the VISTOS unit of an SRYC facility, where she had resided for 15 months.

### Participants

Seven semi-structured interviews were conducted: four interviews with treatment staff (mentor, psychologist, psychiatrist, and a group worker), two with peer support workers, and one with Rose. The first author personally recruited all participants through face-to-face interactions and employed a purposive sampling strategy to ensure a diverse range of perspectives (Mays & Pope, 2000). The female psychologist, 41 years of age with 19 years of work experience, held a university degree. Similarly, the psychiatrist, a male aged 44 with nine years of work experience, also possessed a university degree. The mentor, a female aged 24, had three years of work experience. The group worker was a male 52 years of age, with 22 years of work experience. Both held social work degrees (vocational levels). The male peer support worker, aged 27, possessed six years of experience as an experienced expert. Moreover, the female peer support worker, aged 35 years, accumulated 13 years of work experience as an experienced expert. Both peer support workers held social work degrees (vocational level). The sample adequately represents the proportion of professionals involved in the intervention.

### Procedure

Interviews were conducted exclusively by the first author. Before the interviews, the respondents received an information letter that provided a clear explanation of the purpose and content of the study. Written informed consent was obtained from all the participants. As Rose was over 16 years of age at the time of the interview, parental or guardian permission was not required. An interview guide was developed based on relevant peer support literature and refined through consultations with peer support workers, which consisted of open-ended questions aimed at exploring the essence of the peer worker role. The researcher also encouraged participants to address any concerns they deemed important regarding Rose’s suicidal behaviour, and follow-up questions were posed to elicit in-depth data. All interviews were audio-recorded, transcribed verbatim, and anonymized. The audio recordings were subsequently deleted to ensure anonymity. The average duration of the interviews was approximately one hour, with the interview involving Rose lasting nearly two hours. No dropouts or refusals occurred during the study period. All interviews were conducted face to face at the SRYC institute. During the member check, all participants confirmed the accuracy of the transcriptions, rendering repeated interviews unnecessary. The manuscript was sent to Rose and the treatment team. There were no objections for publication. This study adhered to the guidelines outlined in the Consolidated Criteria for Reporting Qualitative Research (COREQ; Tong et al., 2007). According to the Medical Ethics Review Committee Arnhem-Nijmegen, our study was not subject to the Medical Research Involving Human Subjects Act; thus, no official approval of the committee was required (2023–16376).

### Reflectiveness of the main researcher

The interviewer (first author, female) was 56 years old at the time of conducting the interviews and had extensive experience interviewing both suicidal adolescents and professionals engaged in SRYC. Her previous work recommended including peer workers in the usual care at the SRYC, which led to this intervention and to the present study. Consequently, she established interactions with the participants before the interviews. Owing to the phenomenological research approach employed, the interviewer consciously set aside personal preconceptions and biases to approach the data with an open mind. This process, known as “bracketing”, involves temporarily suspending personal beliefs and assumptions (Charmaz, 2014; Smith et al., 2009). The interviewer engaged in reflective practices that were facilitated by in-depth discussions during various research meetings to support this approach.

### The intervention

At Rose’s request, peer support workers were added to care as usual. The peer support treatment was newly introduced to the facility, and as such, there was no opportunity to offer Rose this treatment during the first 15 months of her care. This limited the ability to provide the intervention earlier in her treatment journey. The peer support intervention was conducted weekly for one year, spanning from September 2021 to September 2022. The duration of each session was flexible and tailored to the specific needs of Rose, with an average length of one hour. The content of the conversations was collaboratively determined by Rose and peer support workers. Peer support is not based on psychiatric models, diagnostic criteria, or predetermined treatment plans. Instead, it primarily focuses on empathetically understanding Rose her situation through the shared experience of emotional and psychological pain (Mead et al., 2001). No treatment goals were established prior to intervention. During the weekly sessions, peer support workers discussed various matters with the young person. Emotional support features listening and talking calmly, being available, and staying nearby. The peer support intervention consisted of two individuals who personally received residential (mental) youth care and subsequently pursued higher vocational education after discharge. In addition to formal education, both peer support workers completed certified peer training facilitated by peer workers. As professionals, they possess extensive firsthand experience in offering inpatient and outpatient support to young individuals facing mental distress by utilizing their experiential knowledge. Throughout the intervention, peer support workers were engaged as experienced experts on a self-employed basis, and were not formally employed within the workforce of the SRYC facility. During the intervention period, four evaluations were conducted, involving Rose, two peer support workers, a psychologist, a psychiatrist, the mentor and the interviewer. The evaluations aimed to assess the benefits of the intervention and involved brainstorming, planning, implementing, and reflecting on its progress.

### Description of analysis

All seven interviews were analysed using an inductive, data-driven thematic analysis approach (Braun & Clarke, 2006), which was aligned with the adopted phenomenological approach to data analysis (Ho et al., 2017). The transcripts were analysed using Atlas.ti version 8.4 (Scientific Software Development GmbH, Berlin, Germany). Two bachelor’s students, referred to as student researchers, participated in various stages of the project as a part of their research course. They assisted the first author in transcribing, coding, and analysing the interviews. Additionally, the analysis was conducted in collaboration with a peer support worker who specializes in research and has experience in delivering peer support to young people aged 12–18 years, both in inpatient and outpatient settings. It is important to note that this peer support worker was not directly involved in the intervention. The analysis process involves iterative and recursive steps, moving back and forth between the data and identified codes (Braun et al., 2019). After familiarizing themselves with the data through repeated readings, the first author and two student researchers independently coded each interview. The codes were then compared and discrepancies were discussed to establish inter-coder reliability (Braun & Clarke, 2006). Intense discussions and conversations occurred among the researchers, and the peer support worker provided additional insights based on her own experiences (Glaser, 2002; Glaser & Strauss, 2008). The initial coding (Saldaña, 2016) of all the interviews resulted in 490 individual codes. The next step involved grouping codes that addressed the same issues into 180 codes, resulting in three main themes with several subthemes (Charmaz, 2014). In addition to the interviews, four evaluations, participant observations, email exchanges, and telephone conversations were conducted with participants to ensure clarity and triangulation of the data. During these moments, the researchers kept notes, which were later elaborated upon, resulting in further clarification of the topics discussed. Furthermore, the research team discussed the findings, and conversations with critical friends from the Karakter Academy Research Department enriched the discussions on power dynamics, ethics, responsibilities, and role clarity. Finally, the analysis phase included a review of the data to ensure sufficient support for each theme and to extract direct quotes that exemplified the themes and subthemes. The use of investigator triangulation involving multiple researchers contributes to the dependability and confirmability of the study (Creswell, 2018). A research diary was maintained to document important steps and changes made throughout the research process. In qualitative research, rigour pertains to the level of trustworthiness and credibility of research findings (Zuidersma et al., 2020). Several methods have been employed to ensure rigour. Prolonged involvement was practiced, which entailed spending a year developing a deep understanding of the context, the work of professionals, and the experiences of peer support workers and Rose.

## Results

**Figure 1.**
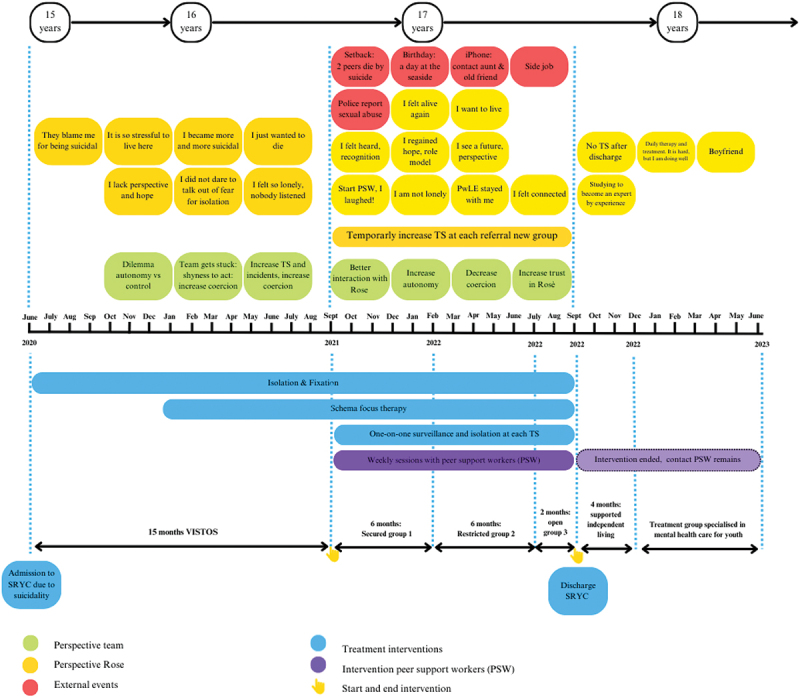
Rose’s patient journey.

This study focuses on the effects of a one-year intervention with peer support workers on a chronically suicidal adolescent residing in a secure residential youth care facility in the Netherlands. Moreover, we explored the mechanisms that underpin the role of peer support workers in detail. Before we begin the Results section, we provide a short description of Rose’s patient journey (see Figure 1). After her admission to the SRYC, her suicidal behaviour worsened, and staff increasingly responded with coercive measures, which negatively impacted her, as well as the therapeutic relationship with staff members. Rose made multiple suicide attempts on a daily basis. She had no contact with family or friends. She lost hope and felt lonely. Therefore, she requested to speak with a peer support worker. As the suicide risk escalated and the situation became unmanageable, the treatment coordinator and psychiatrist decided to grant the request and turned to peer support workers for help. Their consideration to do so was driven by feelings of helplessness and the staff’s fear of a fatal incident (risk management). Rose described her feelings before the start of the intervention as follows.

If I attempted suicide, I was being put in solitary confinement. It felt like being punished because it felt as it was all my fault, while in reality, I just needed help deeply [cries]. Being isolated makes one want to die even more. I felt an overwhelming sense of loneliness and isolation. I longed to talk to someone who had experienced struggles similar to me, but who had successfully overcome them. Someone who had the knowledge and experience to truly understand what I was going through. (Rose, 17 years)

The results were organized across three main themes: 1) mechanisms that underpin peer support intervention, 2) the effect of peer support intervention, and 3) barriers and facilitators. The themes were divided into subthemes, as shown in Table 1 and discussed separately below. The quotes were translated from Dutch.

**Table 1.** Overview of the themes and subthemes.

Theme 1: Mechanisms that underpin the peer support intervention	Theme 2: Effect peer support intervention	Theme 3: Barriers and facilitators
Subtheme 1.1: Emphasis on relation	Subtheme 2.1: Decreasing effect on suicidality	Subtheme 3.1: Barriers
1. Developing a meaningful relationship 2. Embracing recovery: a shift in perspective towards suicidal behavior 3. Risk object versus a young person in need of help 4. Risk management through active listening 5. Unleashing potential: embracing strengths instead of problems 6. Recognition and role-modelling: providing hope 7. Lived experiences as a creative source		1. Role confusion due to lack of communication 2. Lack of organizational facilitation. 3. Fear for criticism 4. Lack of knowledge concerning recovery related care
Subtheme 1.2: Practical support	Subtheme 2.2: Educational impact on staff	Subtheme 3.2: Facilitators
1. The mobile phone as a gateway to the world 2. Nurturing continuity: providing consistent support and stability 3. Activating as a source of recovery 4. The bridging role: empowering, advocacy		1. Act first, think later 2. Unlocking collaboration through professional Expertise 3. Time as a catalyst
	Subtheme 2.3: Disturbing effect on peer support workers	

## Theme 1: mechanisms that underpin the peer support intervention

### Subtheme 1.1: emphasis on relation

#### Developing a meaningful relationship

At the start of the intervention, the two peer support workers focused on building “ordinary” connections with Rose through connecting with her in daily life situations, showing genuine interest, and listening sincerely. Using their own lived experiences with suicidality and youth services, they established trust-based relationships with Rose more easily than traditional youth-care workers. By sharing their personal experiences, the peer support workers helped Rose feel understood and “normal.” This shared understanding and direct, honest, and warm conversations created a safe space for Rose. She described her first experience with the peer support workers as a turning point, where she felt happiness and joy for the first time in a long period, breaking the deadlock in her treatment and feelings of being misunderstood.

Rose recalled this moment lively.

And I remember when I met Steven and Christy. I was going through a really tough time. I was so sad. I had not laughed for a long time. … And then, I had visiting hours with Steven [laughs heartily], and we had these little plastic sticks to stir tea, you know. We then started making bows and arrows, and we had bow and arrow fights. And I remember that I laughed very much. I was literally crying and laughing for an entire hour. Just laughing and it felt so good and relaxed. It wasn’t described in any treatment plan, like, ‘you must make have a bow and arrow fight with stir sticks.’ No. He did something at that moment. I laughed so very much, and I am still grateful to him. I just came alive again. (Rose, 17 year)

All participants noted that by connecting and establishing a meaningful relationship with Rose, peer support workers broke down her isolation.

*…* because she was really very lonely … she did not have any contact with her family or friends … .she had nobody to turn to. The peer support workers made a connection with her. She was not that lonely anymore. (staff, male)

Building a trusted relationship sounds simple; however, a great amount of effort is required to truly listen and build such a relationship.

You really have to make an effort. This is not something that simply happens by joking around or being silly. You have to handle this with great awareness. You really have to make contact, really listen, really look, see, and feel. (p*eer support worker, male)*

#### Embracing recovery: a shift in perspective towards suicidal behavior

The peer support workers approached Rose from a recovery-oriented perspective, focusing on understanding the reasons behind her suicidal behaviour, unlike the staff’s behavioural approach that mainly managed risk factors. By listening and drawing from their own experiences, the peer support workers viewed Rose’s suicidality as a normal reaction to her challenging circumstances and provided a supportive environment. They openly discussed difficult topics, such as the deaths of Rose’s former groupmates, which the staff often avoided due to fear of setbacks. Rose conveyed that she often felt like a burden to the staff. The peer support workers reassured Rose that she was not a burden but rather valuable, and that they genuinely cared about her well-being. This confrontational approach effectively challenged her perception of the burdensomeness. Furthermore, the fact that suicidality was not taboo created a space for her to explore life and no longer desire immediate death. Rose felt that she was no longer alone in the world as she found people who stayed with her during crises and expanded her interactions with the world around her.

Peer supporters work more with recognition and empathy. They did not give standard responses. It is much more like ‘I understand you, and I have been through something similar,’ and then you start talking about each other’s experiences and find a lot of recognition. It is much more about being together, sharing, and recognizing. They listened to me. I did not felt lonely anymore, I just felt accepted and cared for. (Rose, 17 year)

#### Risk object versus a young person in need of help

The staff emphasized that to manage the suicide risk posed by Rose, a variety of mechanisms were employed, with a particular emphasis on implementing coercive measures. These measures included solitary confinement, camera surveillance, and restrictions on mobility, all aimed at ceasing her suicidal tendencies and preventing Rose from causing harm to herself. The use of coercion was underpinned by a firm belief in the importance of risk management, which was deemed logical and necessary by the staff involved. According to the peer support workers, the staff’s therapeutic engagement with Rose and discussions about suicidality were replaced by a focus on risk management. At times, Rose was seen as a risk object, with risk factors and deficits, rather than a young person needing help. This view highlights the contrasting roles of peer support workers in this context as observed by both staff and peer support workers.

It is true that we react to and focus on her suicidal behavior. We have little time to sit calmly with her and discuss her feelings. We also have a different assignment, ensuring that she does not die. And if she attempts suicide, we ensure she does not succeed. It may sound a bit harsh and very unpleasant, but children do die here. This must be taken into account. It simply happens in SRYC. Despite everything we do to prevent it. Therefore, we must deal with this risk. And the peer support workers do not have to do so, they just stand beside her. They are simply humans in their interactions, and listen to her. They offer something we cannot offer. (staff, male)

While the implementation of coercive measures aimed at mitigating risk, Rose’s suicidal tendencies persisted and intensified over time. This escalation of Rose’s suicidality occasionally left the staff feeling helpless and impeded their ability to effectively listen to and understand her situation. Repeated exposure to Rose’s suicidal behaviour caused a range of reactions in the staff, including fear, anger, and frustration. Additionally, this led to emotional numbing and diminished empathy among the majority of staff, which obstructed their therapeutic relationship with Rose.

I once said to her: “Well, what do you expect from people if you keep hanging on a rope all the time? How do you think that makes us feel? People are shocked and broken down by what you do! Just stop behaving suicidal and take control of your life”. (staff, female)

And yes, there’s also a kind of irritation that comes with it. It is not pleasant to constantly see someone hanging with a rope around their neck, as Rose often did. Yes, it’s really very stressful; you have to cut her lose, just thinking about how to save her life. At such moments, I could not listen to or talk to her. I am only human; I could not. I was too disturbed myself. (staff, male)

#### Risk management through active listening

Remarkably, both Rose and peer support workers emphasized that, in general, they did not overly talk about Rose’s suicidality. Instead, they discussed other factors that caused her suicidal feelings, such as difficult situations with group leaders, Rose’s struggle to communicate her feelings, loneliness, and sadness about her personal family situation. In contrast to the staff, peer support workers listened to Rose’s suicidal feelings without any physical intervention to protect her. Whenever Rose experienced a crisis, peer support workers remained present and maintained calm demeanours. In doing so, they co-regulated Rose’s heightened suicidal emotions. The peer support workers viewed Rose as a young person facing difficulties, which she expressed through her suicidal behaviour.

I do not focus on the suicidal behavior at all. And I do not see her as a patient. I just meet with her as a human being, and I look beyond the suicidal behavior. If you do so, you can understand her more easily. I listen to her but do not get upset or angry when she is suicidal. I make contact with her based on my experience. I accept her suicidal feelings and stay calm. That calms her down. You know, that’s a whole different approach than saying to her, “You’re not allowed to behave suicidal, so don’t do that!” (peer support worker, female)

#### Unleashing potential: embracing strengths instead of problems

The peer support workers supported Rose by focusing on her strengths rather than problems, creating a safe, non-judgemental space for her to express herself. They emphasized active listening, understanding her suicidal behaviour, and building a genuine connection through ordinary, agenda-free conversations. Their weekly meetings focused on Rose’s current situation and life experiences. Initially, Rose felt hopeless and saw no future, but the peer support workers maintained a positive outlook and highlighted her abilities. They discussed her future and shared examples of other young people with similar backgrounds who had successfully reintegrated into society. This approach helped Rose to see her own potential and consider possibilities for her future, countering her sense of hopelessness and feeling lost.

What I also did was emphasize that she can pursue an education in the future. Sure that you can achieve something meaningful in your life. What the F*ck. Why would this not be possible? Who says you can’t? She started this negative thinking that it was impossible because of the situation she was in and the heavy stuff that had happened to her. However, I told her about all the examples of young people who had a similar background, but who found their place in society in various ways. However, if nobody tells you that, you just feel lost. And you end up believing that you never will get any further. (peer support worker, male)

#### Recognition and role modelling: providing hope

Both the peer support workers shared their experiences with Rose. They adjusted their approach to contribute to the situation in which Rose found herself at that moment, ensuring that their input would be helpful for her. Some staff described the peer support workers as role models or “living examples of hope” Rose felt that talking with the peer support workers about their own recovery and ability to function well socially gave her a sense of hope for her own future. This made her feel less lonely, knowing that she was not the only person who dealt with these problems. She was not “strange” anymore. Rose stated that one helpful aspect of the intervention with peer support was “talking to someone who has felt the same.” Moreover, peer support workers are outside the SRYC system. Rose felt comfortable discussing the system with someone she trusted, who was familiar with it but had moved on from where Rose currently saw herself. Sharing these experiences ultimately promoted hope for Rose and seemed to underlie the role-modelling effect: the instillation of hope through positive self-disclosure, which Rose described as follows.

Steven (male peer support worker) also spent time in SRYC. However, he is ‘healthy’ now, has a great life, and even works in the healthcare field. Knowing that he managed to overcome his problems that truly helps. Christy (female peer support worker) is also a role model for me. She has dealt with the same issues as me, and when I see her, I realize [with a surprised tone]: I can really overcome my issues! Because she is pregnant, she will soon have her own family. And that … I just felt like ‘Wow, that might be able for me too!’ That really gave me hope. (Rose, 17 years)

#### Lived experiences as a source of creative solutions?

Experiential knowledge was described by all participants as an additional source of knowledge that played a crucial role in peer support worker relationships. Peer support workers brought a wealth of personal experience and knowledge, which created a broader and more diverse perspective on Rose’s problems. According to Rose, they do not rely on protocol-based approaches. Instead, they offered a wide range of options and creative alternatives in which they focused on possibilities instead of limitations. They provided Rose with more creative solutions that expanded Rose’s perspective on what was possible and opened up new horizons for her. According to Rose, the practical and emotional support of the peer support workers empowered her, making her feel accepted and included. The trusting relationship with the peers support workers also influences Rose’s other relationships and social capital. Feeling connected provided her with hope and made her feel less lonely. According to her, weekly contact with the peer support workers saved her life.

They have experienced so much, which has given them a different kind of knowledge. Thus they have a broad perspective. They supported me by taking steps forward and kept saying that they believed in me. They often thought outside the box to help me with the challenges I was facing, which gave me new insights and, above all, perspective! You know, they really have saved my life. (Rose, 17 years)

### Subtheme 1.2: practical support

#### The mobile phone as a global gateway

Upon admission to SRYC, the standard procedure is to confiscate the youth’s phones. Hence, Rose did not have access to internet or social media. Recognizing the importance of connecting with the outside world the peer support workers ensured that Rose obtained a mobile phone. This seemingly small gesture had a significant impact on Rose’s life. Having a mobile phone allowed her to catch a glimpse of the world beyond SRYC and opened opportunities for education and employment. The peer support workers challenged the notion of limitations and encouraged Rose to focus on her strengths and opportunities. Moreover, having a mobile phone enabled Rose to contact the peer support workers independently during times of crisis. It also allowed her to reconnect with her aunt and best friend. Activities, such as going for a weekend trip and finding a side job, contributed to these outcomes. This approach had a positive effect on her self-esteem and self-efficacy, and therefore, increased Rose’s social inclusion in real life. Initially, peer support was only available in person. However, with the introduction of the mobile phone, support was extended to telephone and WhatsApp. This meant that Rose could reach out for help whenever needed, increasing her ability to manage her difficulties effectively.

Well, if I’m completely panicking, I can call Steven or Christy. I can just call them first. At a certain point, I had an iPhone for a few hours a day. I could call them and ask for their help. I could rely on them. (Rose, 17 years)

Initially, the group leaders faced significant difficulties when Rose obtained a mobile phone, especially because the institute’s policy prohibited the rest of the youth from holding mobile phones. Additionally, the group workers were concerned that Rose if would receive a phone, while the rest of the young people did not, this would lead to non-acceptance within the group. This situation created tension and presented a challenge to staff members, who were also concerned about risk management.

Yes, that iPhone situation was quite challenging. For, how do you position yourself in this situation, and how do you sell it to the rest of the group? In addition, there was a lot of distrust that we acquired over time with Rose. Like, hoping she does not commit suicide, or things get worse if she starts calling strangers. You start imagining all sorts of things. (staff, male)

To mitigate the risks associated with phone use, the facility implemented several safeguards, including regular check-ins with Rose about her phone use, limiting her access to specific times, and closely monitoring her phone use by the treatment team.

#### Nurturing continuity: providing consistent support and stability

The peer support workers played a significant bridging role among adolescents, staff, and formal institutions. They leveraged their positions to support Rose by communicating effectively with the staff. Operating at the boundary between professionals and Rose, peer support workers bridged the gap and facilitated a deeper understanding of Rose’s behaviour for the staff. Over the course of one year, Rose underwent several transitions, moving from a very high intensive care unit to a closed unit, then to a restricted unit, followed by an open group, and ultimately to discharge. During each transition, Rose was confronted with a new mentor, new group leaders, and interactions with new peers. Consequently, each step poses potential disruption, causing significant stress and anxiety. Confronted with constantly changing healthcare providers confirmed her feelings of being burdensome, unmanageable, and hopeless. This resulted in an increase in suicide attempts before, during, and after each transitional period. Therefore, the peer support workers remained a constant source of support for Rose throughout these challenges, providing assistance during her journey in SRYC (and beyond). Their unwavering presence offered continuity over time. As a result, the peer support workers were more likely than other professionals to develop a strong emotional connection with Rose. Walking alongside her throughout her recovery journey, they conveyed a message of care and involvement that alleviated Rose’s feelings of isolation, burden, and loneliness. Despite the persistence of her suicidal behaviour and the added stress of transitioning within the institution, the consistent presence and support of the peer support workers throughout the one-year intervention played a crucial role in her path towards recovery.

You know, the peer support workers came every week and yes, that was really one of my rescues. Both of them understood me. I do not have many people who understand me, and the youth care professionals are temporary. They go away. When I go to a new group, they do not stay with me. My peer support workers just stay with me. (Rose, 17 years)

#### Activating as a recovery source

During the peer support intervention, peer support workers engaged in various activities with Rose. Although these activities seemed like small steps, the peer support workers made time to go for walks with Rose, which the group leaders often did not have time to do. Being away from the group proved beneficial for Rose and these activities gradually expanded over time. Being outdoor and engaging in activities positively influenced Rose’s mental well-being and self-confidence. As a result, Rose started experiencing a heightened sense of vitality and overall improvement. A significant moment occurred when the peer support worker took her to the seaside on her birthday, providing her with an opportunity for enjoyment and creating positive memories. Prior to this, Rose had not experienced any outings outside of the SRYC facility.

At VISTOS, I was kept indoors for one and a half years, for whenever I had a chance to be outside, I used it to run away because I desperately wanted to get away from SRYC. At some point, Steven and Christy took me to the outside. That activation, - just doing fun activities in society -, helped me a lot during that time. I went to the beach with Steven (male peer support worker), and I often went for walks with them. These moments were very enjoyable and contributed to my regaining trust in being in the ‘normal’ world, knowing that I had to break free from the world of SRYC. And that it could get better. In that sense, being activated in the outside world was very beneficial for me. (Rose, 17 years)

#### The bridging role: empowering and advocacy

The peer support workers served as a bridge to facilitate Rose’s transition to new groups, making it easier for her to provide stability and support. Although the peer support workers initially needed to establish legitimacy with the members of the treatment staff, this gradually changed over time. At a certain point, one of the peer support workers participated in one of Rose’s therapy sessions. The treatment had reached a standstill because of the resistance of Rose to the therapy. During the therapy session, the peer support worker provided subtitles and clarifications for both Rose and the therapist, effectively bridging the gap between the youth and therapist, while sharing their unique perspectives. Thus, the peer support worker paved a trust-based pathway for the therapist to convey her intentions to Rose. This resulted in both parties gaining an understanding and a better comprehension of each other, ultimately breaking the treatment impasse.

I remember a session with Christy, Rose and myself, where the peer support worker took on that bridging role. What I had been trying for 10 months with Rose, the peer support worker managed to accomplish in one session. She clarified my position to Rose and enabled Rose to open up and be vulnerable to me. That was, of course, very challenging for Rose. After all, I had the authority to isolate her when she was suicidal. The session was a beautiful and significant experience. Also very emotional, both for me and Rose. (staff, female)

The peer support worker supported Rose to take more control over her life, increasing Rose’s participation in her own illness management by being present and encouraging her to speak her mind during meetings. Rose wanted treatment for her trauma’s, which could not be provided for in SRYC, as Rose would turn 18 soon. However, Rose and staff experienced several barriers during the transition between SRYC and adult mental health services. At some point, it became evident that there were several issues with enrolment procedures at the new institution. Additionally, the municipality was unwilling to provide funding. It seemed that Rose could not transition to a follow-up location for treatment. Rose was very upset, and the uncertainties about the next therapy group after discharge caused Rose to panic and become very angry with the staff. The peer support workers managed Rose’s expectations and assured Rose that the staff members were credible and trustworthy. Through their presence and the rapport they built, Rose placed her trust in the peer support workers, allowing her to listen to her treatment staff. At the same time, peer support workers helped the staff facilitate a hopeful and timely transition. In short, peer support workers played a bridging role in facilitating the transfer to a suitable treatment facility.

Yes, I stood up for her. I said, “I don’t care, but I will be present when this is discussed in a meeting.” And I made it very clear: I said, ‘If you do not organize this transfer, then I will see what I can do. I will make some calls and we will plan an introduction to an alternative location to see if Rose can be accommodated there. We will arrange this properly, and I am more than willing to utilize my knowledge and network for that purpose, which I did. (peer support worker, female)

## Theme 2: the effect of peer support intervention

### Subtheme 2.1: decreasing effect on suicidality

All participants agreed that Rose’s suicidal tendencies decreased during the peer support intervention, although her suicidality did not completely disappear. Stressful events, like moving to a different group or the suicide of group members, still triggered her suicidal thoughts. However, Rose’s help-seeking behaviour improved, as she increasingly sought contact with the peer support workers and shared her suicidal feelings with them. The trusting relationship Rose developed with the peer support workers positively influenced her previously difficult relationships with the treatment team, reducing conflict, tension, and stress. Over time, as Rose’s suicidal behaviour decreased, the staff placed more trust in her, granting her more autonomy, which correlated with fewer suicide attempts. While the group leaders recognized the positive impact of the peer support workers, some team members were sceptical about attributing the reduction in Rose’s suicidal behaviour solely to their involvement. Rose’s psychiatrist and therapist acknowledged the vital role of peer support in granting Rose more autonomy and trust but noted the difficulty in establishing a clear causal link.

What I have definitely observed in the period after the introduction of the peer support workers, is an improvement in the collaboration between her and the treatment team. This progressed slowly with ups and downs. Rose’s self-destructive behavior gradually decreased in frequency and severity, and the team could implement a more autonomy-promoting policy. I believe that the involvement of peer support workers certainly contributed to this, although establishing a causal relationship is difficult, of course. (staff, female)

Rose described how her recovery from suicidality began with the support of the peer support workers. The simple act of being heard and listened to served as a catalyst for Rose to open up and discuss her suicidal feelings. At the same time, Rose gained new perspectives, as the peer support worker consistently inquired about her desires and life goals. The acceptance and acknowledgement from the peer support workers made Rose more receptive to their suggestions compared to the advice from the staff. This support helped Rose regain hope and perspective, leading to a reduction in her suicidal thoughts and behaviours. Rose highlighted the significant and positive impact of the peer support workers throughout her recovery journey.

They […….silence…….] have meant so much to me. They gave me recognition and acknowledgment of what I was feeling, and above all: Perspective! And yes, that is, just a different level of support. Since I have them [peer support workers SK], I have grown so much. They really helped. I started to feel better. I think they are the main reasons that I am still alive today. (Rose, 17 years)

### Subtheme 2.2: educational impact on staff

The intervention was initiated when the conventional treatment had reached a deadlock, and there was a pervasive fear of losing Rose. Implementing this intervention and observing its effectiveness not only instilled confidence but also had a profound educational impact on staff. Moreover, the peer support workers were able to illustrate the possibility of recovery for suicidal adolescents with whom they worked, and they increased hopefulness and optimism across the teams in which they were based. While the staff anticipated some positive results, they were surprised by the extent of the intervention’s benefits. The outcomes exceeded the staff’s initial expectations, and yielded far more positive results than anticipated.

I did not anticipate that it would have such a profound impact on all of us. What is somewhat overlooked is that chronic suicidality can destroy entire teams. And with Rose, the team was truly on the brink of collapse. Through the peer support worker, we were able to create a fresh start. This result exceeded our expectation. I learned a lot. Thus, the intervention has been valuable in many ways. Personally, I regained my sense of calm because I saw how helpful it was. (staff, male).

Peer support workers served as role models for recovery in two contexts: interacting with staff members at the SRYC and supporting Rose. Some staff members gained valuable insights from the peer support workers, particularly in how to approach suicidal adolescents with increased empathy and understanding. This led the staff to recognize the importance of forming deeper connections with suicidal adolescents in their daily work.

They forced me to work as a human again. They brought me a lot in terms of not alienating from our patients and focusing blindly on the system. They brought back the human scale. (staff, male)

### Subtheme 2.3: disturbing effect of SRYC on peer support workers

The interviews revealed that working in SRYC had a significant emotional and professional impact on both peer support workers. They expressed frustration over the staff’s lack of knowledge about suicidality and self-harm, which led to a reliance on behavioural interventions rather than exploring underlying issues. The peer support workers were at times distressed by the coercive measures used on Rose, which reminded them of their own past experiences with similar treatment. They felt that Rose needed connection and support rather than isolation and coercion. However, it was too early for them to voice their criticism of the coercive measures applied. Nonetheless, at times, the emotional impact of working in SRYC weighed heavily on them and made them feel powerless, angry, and sad.

I have often left the SRYC facility with stomach pain, tears in my eyes, feeling overwhelmed. I would often need to call friends while driving home, expressing my frustration and anger and venting my sadness about the kids I had to leave behind there. The way these kids are treated is not helpful. How are these kids supposed to feel better in such sick environments? Surrounded by fear and these messed-up coercive measures? It’s bizarre. And Rose is not alone; there is an entire group of young people who experience this. I often felt powerless and angry. It is incredibly sad how things go. It’s not easy to deal with. (peer support worker, male)

Both staff and peer support workers faced significant worries and frustrations when struggling to secure a suitable follow-up placement for Rose after her discharge. Her persistent history of suicidal tendencies made finding appropriate continued care challenging. As the discharge date approached, uncertainty and stress increased, leaving both Rose and the peer support workers feeling powerless. They recognized the risk of patients falling through the cracks in the healthcare system, which could heighten suicide risk. They also felt inadequately prepared to support Rose through her transition due to the lack of a finalized placement until the last minute. Throughout this period of uncertainty, peer support workers witnessed how Rose experienced heightened vulnerability and elevated suicide risk. Nevertheless, both peer support workers felt that they were not in a position to voice their criticism of systemic failures at this particular juncture.

My priority at this moment is to build a strong rapport with the staff and demonstrate our value. It is still too early to express criticisms. If I were to do so, I would risk being immediately sent away, which would hinder our ability to have a positive impact on these kids. Therefore, I choose to keep my thoughts to myself. (peer support worker, female)

## Theme 3: barriers and facilitators

The final theme focused on factors that facilitated or obstructed the implementation of peer support interventions in practice. Inevitably, the intervention with peer support workers did not proceed smoothly. There were a range of challenges surrounding the introduction of peer support into the existing structures and culture of practice within the SRYC.

### Subtheme 3.1: barriers

#### Role confusion due to lack of communication

The group workers noted that the introduction of peer support workers was not communicated in advance, leading to confusion about their roles and responsibilities. They felt there was insufficient clarity on how peer support workers should collaborate with the treatment team and whether they would contribute to treatment goals. Concerns were raised about professional boundaries and the extent of information that could be shared with peer support workers. While it was recognized that peer support workers had different responsibilities than treatment staff, there was significant uncertainty and distrust about how their role would integrate with SRYC services.

I don’t know exactly what their job description states, I did not know what they were supposed to do here. What experience do they have? One day, they were there, and we were surprised and annoyed. Yes, there was an email, but that was it. We did not know what to expect. So that was not very helpful in terms of collaboration and teaming up with the peer support worker. (staff, female)

#### Lack of organizational facilitation

Initially, problems concerning the payment of peer support workers were resolved over time. There were additional practical issues that needed attention: Group leaders were often unaware of the arrival of peer support workers. If an appointment was not recorded by the group leaders, it could happen that Rose was not present in the group, and the peer support worker arrived for nothing. This led to frustration and annoyance for both peer support workers and Rose. When Rose’s condition improved, she was transferred to another group with more freedom. However, with each transition to a different group, the same practical issues regarding scheduling appointments have emerged. Moreover, the teams in the new groups seemed to be uninformed about the intervention and, at times, the peer support worker felt undervalued.

Rose was often moved between different groups on the premises and received frequent one-on-one support. It became frustrating for me to introduce myself every time to a different person, you know? Who am I? What am I here for? It always feels like I have to justify myself because it is something they are unfamiliar with. Furthermore, there exists a certain level of tension, and individuals may have reservations about working with us. They wonder, “Are you one of those young people who have been here too?”. (peer support worker, male)

#### Fear for criticism

As previously described, most staff members initially recalled reservations about working with peer support workers. One staff member explained that she feared criticism. However, over time, these fears disappeared as she recognized the potential for improved treatment outcomes by working with a peer support worker. This shift in perspective helped the staff move from feeling irritated and exhausted by Rose’s suicidal behaviour to developing increased confidence and trust in their ability to handle the situation effectively.

At start I felt uncertain myself. I was afraid to be criticized. I myself also know that there are many things wrong with what we do here, and I did not feel like hearing it all over again in great detail from my peer expert. However, thankfully, the opposite was true. They were constructive and supportive, and we regained faith in supporting Rose. All my concerns turned out to be much less significant than I anticipated. (staff, female)

#### Lack of knowledge of recovery-related care

Moreover, professionals sometimes lack a sufficient understanding of the value of lived experiences for adolescents in SRYC, resulting in resistance to working with peer support workers. Part of this resistance stems from unfamiliarity with recovery-related care and the role of peer support workers. Although staff resistance was still present, both peer support workers began to address this issue over time by introducing themselves during their visits to Rose. They approached these interactions with an open and positive attitude, demonstrating their willingness to support the staff and facilitate collaboration, rather than burden them. However, upon entering the youth’s room and seeing Rose texting with her peer support worker instead of communicating with her, one participant felt a mix of envy and anger.

I entered her room unexpectedly and found her on the phone with her peer support worker, without me knowing beforehand. At that moment, I started to feel a sense of deception or something similar. It troubled me that I could not fulfill my role, as the young person solely sought out the peer support worker and no longer approached me. This made me question whether it was still appropriate to allow her to continue contacting them. (staff, female)

Rose explained why she choose to confide in her peer support worker rather than her mentor:

I do not talk to my mentor about my suicidal feelings. If I share my suicidal thoughts, she isolates me, she does not understand. This made me feel more miserable. Therefore, I remain silent and prefer to talk to my peer support worker. I want to be understood, not judged. They listen and that helps me to feel better. (Rose, 17 year)

### Subtheme 3.2: facilitators

#### Act first, think later

The intervention was developed and guided by a dedicated team committed to creating a recovery-focused approach for Rose. They embraced a growth mindset, recognizing that integrating peer support workers into SRYC was a new and challenging process. The team, starting without a pre-conceived plan, focused on building quality relationships among staff, peer support workers, and Rose. They emphasized dialogue and transparency through regular meetings and fostered trust. Over time, staff perceptions of peer support improved as discussions with peer support workers and Rose addressed concerns and fears. For example, it was exciting for the staff to give Rose more autonomy regardless of her suicidality. The team’s willingness to embrace uncertainties and openly discuss challenges contributed to the intervention’s success.

#### Unlocking collaboration through professional expertise

By working in the field as professionals themselves, peer support workers understood both sides of the coin: youth care professionals and service users. This enabled them to recognize the structure and dynamics that teams in SRYC were experiencing. It appeared to be important for teams to know that the peer support workers also worked as “regular” professionals in order to be more accepted as full-fledged professionals. As soon as the peer support worker mentioned having received the same education and training as the professionals in the team and working as a professional themselves beside their work as a peer support worker, the attitudes of the professionals changed. They were more inclined to see peer support workers as equal, recognizing the value of their professional expertise, as well as their experiential knowledge. This shift ultimately benefited their collaborative work.

I always clarify right away that I work as a professional as well. I have a college degree and, in addition, I bring my own personal experience to my work. It’s interesting because when I mention this, you can see them relax and become more accepting. (peer support worker, male)

#### Time as a catalyst

The duration of the weekly peer support worker intervention was one year. The continuity of support allowed the peer support workers to assist Rose not only briefly but throughout all the steps in her recovery journey. The peer support workers were present at all transitions in SRYC that Rose went through. Over time, this has enabled Rose to gradually get to know and develop a better understanding of the peer support workers, leading to an increased level of trust.

They stayed with me for a longer period. It was so nice. I could talk to them about everything that was on my mind. That really helped me. (Rose, 17 years)

## Discussion

**Figure 2.**
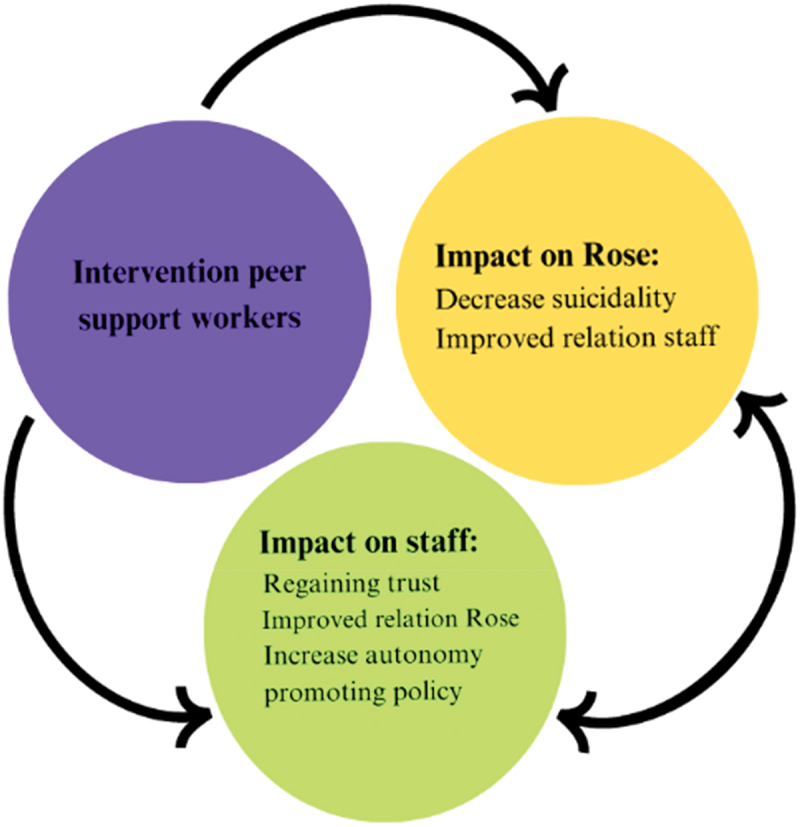
Impact intervention peer support workers: chain of events.

The results show a significant reduction in Rose’s suicidal tendencies during the peer support intervention compared to baseline. This decrease in suicidal tendencies led to increased confidence among youth care professionals, who subsequently granted Rose more autonomy and reduced coercive measures. These changes positively impacted Rose’s suicidality. This case report is the first to offer a detailed, systematic exploration of this issue through the participant’s first-person account. Figure 2 provides a visual representation of this process.

Additionally, Rose’s help-seeking behaviour improved during the intervention, as she actively reached out to the peer support workers and openly expressed her suicidal feelings towards them. Although establishing a direct causal relationship is challenging using a single-case design, the results indicate that the peer support intervention played a significant role in the observed decrease in the number of tentamen suicides and the decrease in Rose’s suicidal behaviour over time. The results indicate that the lived experience and empathetic approach of the peer support workers provide a valuable counterbalance to the risk-focused paradigm prevalent in SRYC, allowing for a more compassionate and seemingly more effective approach to address Rose’s suicidality. We would like to emphasize that many competent and experienced youth care professionals understand that an excessive focus on risk management can jeopardize the therapeutic relationship. Consequently, the results of this study do not apply to all professional staff teams in SRYC. The attitude of empathy and understanding is not exclusive to peer support workers; indeed it is the case that professionals also adopt this attitude, as evidenced in practice. This paper aims to share reflections and lessons learned in this case study so that subsequent projects can build upon the results of this project in utilizing peer support to enhance and improve the care provided to suicidal adolescents in SYRC and beyond. We use the Interpersonal Theory of Suicidal Behaviour (IPT) developed by Joiner (Joiner, 2005; van Orden et al., 2010) and the Integrated Motivational-Volitional (IMV) model of suicide developed by O’Connor and Kirtley (2018) to interpret the main findings. Moreover, we use the self-determination theory to elaborate on the principles of peer support (Deci & Ryan, 1985).

### You are not a burden, we care

Rose frequently experienced feelings of worthlessness, loneliness and burden. According to the IPT model, Rose’s intense feelings of burdensomeness and loneliness put her at risk for suicidal thoughts and behaviours. The presence of peer support workers, who demonstrated to Rose that she was not a burden to them and valued her needs, improved her self-worth and self-esteem and decreased feelings of loneliness. By decreasing her loneliness, peer support workers fostered a sense of connectedness, which led to a decrease in Rose’s suicidal behaviour. The IMV model of suicide emphasizes the interaction between motivational and volitional factors in the development of suicidal behaviours. The model also emphasizes the importance of protective factors such as social support and coping skills, which may play a role in preventing suicidal behaviour. Motivational factors include the desire to escape psychological pain, whereas volitional factors refer to the capacity to perform suicidal actions.

The peer support appeared to act as a protective factor by offering emotional support, instilling hope, and providing a future perspective, all of which consequently alleviated Rose’s psychological distress. This decrease in distress seemed to weaken Rose’s motivation to escape her suffering through suicidal thoughts or behaviours. Furthermore, the peer support intervention may have influenced motivational factors by reducing Rose’s thoughts on meaninglessness and perception of being untreatable. The results indicate that over time, peer support provided Rose with alternatives to her suicidal behaviour. These alternatives included addressing her emotions, engaging in open conversations, seeking help, and implementing strategies to cope with her emotions such as activation and distraction. In turn, Rose’s decrease in suicidal behaviour increased staff confidence. Consequently, over time, the staff reduced the use of coercive measures, progressively granting more autonomy, resulting in a further enhancement of Rose’s well-being.

### Impact of sharing stories on recovery

The study by Niederkrotenthaler et al. (2022) highlights the positive and protective impact of stories depicting hope and recovery from suicidal crises (Niederkrotenthaler et al., 2022). Consistent with Niederkrotenthaler’s research, the findings in this study suggest that narratives of recovery and resilience are beneficial in helping adolescents overcome their suicidal tendencies. Sharing stories about recovery and resilience can empower suicidal adolescents such as Rose, by countering feelings of hopelessness and despair. Openly discussing recovery from suicide not only breaks the silence surrounding this sensitive topic but also reduces, rather than increases, suicidal ideation, which underscores the importance of being able to talk about suicide (Dazzi et al., 2014; Kaijadoe et al., 2023). Sharing narratives of hope and resilience can inspire others to seek help, reach out to support, and explore suicide alternatives. Ultimately, this reinforces the belief that recovery is attainable, and offers a beacon of hope to those in need.

### Unraveling recovery versus risk

The experiential expertise of the peer support workers not only seemed to benefit Rose but also enabled staff to identify practical alternatives to coercive measures. Before the intervention, the staff often felt obstructed and frustrated in their efforts to decrease Rose’s suicidality, which in some cases led to rejection and even resentment of Rose’s suicidal behaviour. For example, some staff members believed that Rose’s suicidal behaviour was controllable and originated from poor coping skills. Consequently, some staff members held Rose responsible and felt irritated and angry that she could not control her suicidal tendency. In their view, Rose needed to take control, so to speak, to stop her suicidal behaviour, which she was unable to do on her own.

Working with suicidal young people like Rose inevitably poses a dilemma for youth care professionals: is the youth still capable of making decisions or should treatment providers protect the individual from themselves and take control? In the case of Rose, the staff responded increasingly with coercive interventions to address her ongoing suicidal behaviour. By contrast, peer support workers offered empathy, support, and genuine validation during Rose’s suicidal despair. Ultimately, this approach resulted in a decrease in suicide attempts and behaviours in Rose. Thus, the focus of professionals seems to shift from control and restriction to autonomy, competence, and relatedness, which are the basic tenets of the self-determination theory.

It is important to note that an excessive focus on (apparent) risk management is a pitfall that teams can fall into, however, in our view, it is not something that should solely be resolved by adding peer support workers. Professionals also play a role in avoiding this trap. Essentially, we emphasize that for an effective, recovery-oriented approach, it is crucial for the team of professionals to first embrace, integrate, and train in a shared theoretical framework of recovery-related care.

### Enhancing self-determination through peer support

We argue that peer support work is closely related to the principles of the self-determination theory. This theory posits that suicidal individuals have innate psychological needs for autonomy, competence, and relatedness (Tucker & Wingate, 2014). In the interviews, Rose emphasized that she experienced a lack of connectedness. Moreover, she stated that taking over autonomy and control (lack of autonomy) and using coercive measures (e.g., fixation and isolation) harmed her and increased her suicidal feelings. Furthermore, she criticized the lack of future perspectives and hope (lack of competence), which was crucial for overcoming her suicidal behaviour. In summary, based on the findings of this study and in line with previous findings, a lack of these three basic psychological needs may lead to an increased suicide risk in adolescents (Hill et al., 2011; Kaijadoe et al., 2023; Tucker & Wingate, 2014). Peer supporters in this single case made an effort to address these fundamental needs. This can empower individuals such as Rose to regain control of their lives, make autonomous decisions, and develop a sense of self-efficacy. Through the support of peer support workers who experienced similar challenges, Rose gained a sense of competence and belief in her ability to navigate her recovery journey. Additionally, peer support fosters a strong sense of relatedness and connection, resulting in Rose feeling understood, validated, and supported by her peer support workers. By fulfilling these psychological needs, peer support aligns with the principles of self-determination theory (Deci & Ryan, 1985).

## Implications for practice

By involving peer support workers, the care provided to suicidal adolescents residing in SRYC can be enriched with invaluable insights and experiences of those who have overcome suicidality themselves. While the immediate resolution of suicidal behaviour may not be achieved through experiential knowledge alone, its value lies in its ability to contribute to long-term trajectories and a broader understanding of recovery in the face of risks. In addition to providing one-to-one support to suicidal adolescents, peer support workers can support teams and contribute to the need for knowledge on suicide prevention from a lived experience perspective. Furthermore, peer support workers can engage in team meetings and offer training for suicide prevention centred on recovery-oriented care. Therefore, we emphasize the significance of integrating well-trained peer support workers into the existing SRYC framework. It is crucial to ensure the effective implementation of peer support workers within SRYC, which traditionally places strong emphasis on risk management when working with suicidal adolescents. It is vital to pay attention to the intersection between these two worlds (Byrne et al., 2016; Mulvale et al., 2019). Building on this intervention, we recommend the following when implementing peer support workers in SRYC:

- The sustainable approach involves all layers of the organization when adding peer support workers to care as usual.
- Ensure that teams are well-prepared for the arrival of peer support workers.
- Ensure adequate preparation of peer support workers for their roles in SRYC. The findings indicate that the SRYC system can significantly impact peer support workers, underscoring the importance of providing supervision and support.
- Discuss unconscious beliefs embedded in language: “chronic” or “in recovery”, “symptoms” or “experiences”, “limitations” or “challenges”.
- Ensure clear agreement with the teams regarding expectations and collaboration with the peer support workers.
- Approach to the incorporation of peer support workers with an open-minded emphasis on growth. This suggests a willingness to embrace new ideas, learn from the experience, and continually improve collaboration with peer support workers.
- Ensuring proper embedding, payment, supervision, and support for peer support workers.
- Evaluate regularly with all involved (including residents).
- Utilize the practical/recovery stories of residents who have received support from peer support workers to shape the vision of providing care to suicidal adolescents in SRYC.

## Limitations, strengths and methodological considerations

Our findings should be interpreted with caution. First, the analysis is based on a single case. Although this study provided detailed subjective reports from which novel information could be drawn, the single-case design does not permit generalization. On the other hand, while the generalization of case study findings is restricted to the case itself, the concrete knowledge produced by this case study supports the development of peer support expertise on suicide prevention in SRYC and beyond (Flyvbjerg, 2006). Second, an important limitation of our study is the lack of opportunity for robust evaluation processes to be implemented from the onset of the intervention. Although interviews captured the reflections of staff, peer support workers, and Rose following the intervention, the use of pre- and post- analyses would have illustrated the effectiveness of the intervention with peer support workers more robustly. To capture personal experiences and empirical data, we recommend using a mixed-methods approach in future research. For instance, the use of quantitative approaches may lead to findings related to the economic advantages of employing peer support workers (Trachtenberg et al., 2013). Third, a single case study, while offering detailed insights into one specific instance, lacks the scope and comparative analysis needed to account for variability across multiple cases. This limitation restricts the ability to draw broader conclusions or make general recommendations, as the absence of multiple cases makes it difficult to compare and contrast findings across different settings, thereby reducing the robustness of the conclusions drawn (Kazdin, 2019).

This case study has two significant strengths. First, it closely reflects real-life situations and provides detailed information. Second, it offers valuable insights into the effects of peer support on a chronically suicidal adolescent residing in SRYC through firsthand exploration and reporting, thereby generating new knowledge. Consequently, this case study enhances comprehension and provides valuable lessons, thereby challenging the conventional perspectives on care offered in SRYC.

## Recommendations for future research

Several questions remain to be answered to clarify the difference in approach to risk adopted by peer support workers (in which suicidal adolescents are not seen as a “risk object” but merely as a person undergoing a personal crisis) and staff. Hence, more research is needed to shed light on the different relationships between the risk management employed by staff and recovery-oriented care utilized by peer support workers working with suicidal adolescents. Peer support itself can vary in quality in various ways. However, the discussion on this matter is beyond the scope of this article and we advocate that further research and development in this regard are necessary. Further research on peer support workers supporting suicidal adolescents is necessary to facilitate the widespread implementation of peer support workers in SRYC and other residential mental health care settings.

## Conclusion

In conclusion, the utilization of peer support workers in supporting chronically suicidal adolescents at SRYC represents a novel and impactful approach. The use of experiential expertise in this case report demonstrates the value of incorporating experiential expertise in addressing the suicidal behaviour of adolescents within SRYC. The intervention with peer support workers not only benefited the adolescent but also positively affected the treatment team. The peer support intervention went beyond a purely symptom-focused approach and enabled the treatment team to develop trust and grant autonomy to Rose, which enhanced her well-being. Our research underscores the importance of empathetic connections and personalized care in promoting the recovery and well-being of suicidal adolescents. Given its inherent nature, peer support serves as a valuable avenue to address the need for autonomy, competence, and relatedness of suicidal adolescents (Tucker & Wingate, 2014). The findings of this study contribute to the growing recognition of the value of peer support in suicide prevention and underscore the necessity for its continued integration into care practices.

## Take home message

Integrating peer support workers into Secure Residential Youth Care (SRYC) settings may offer a promising and impactful approach for supporting adolescents at risk. An important implication of involving peer support workers is their ability to connect with adolescents on a personal level; their shared experiences foster trust and facilitate open communication, which is crucial for addressing complex issues like suicidality. This approach significantly impacts treatment dynamics by shifting from a purely clinical focus to one that incorporates empathy and personal understanding, potentially leading to more effective support for adolescents struggling with suicidality as well as the treatment team.

## Beyond the intervention

After discharge from the SRYC, Rose’s suicidal behaviour ceased. Having left the SRYC, she is now actively working to address her traumas, pursue personal and professional goals, and build a fulfilling life. By finding a job and boyfriend, Rose established stability and connections in her life. These positive relationships and the sense of belonging they provide contribute to her overall well-being and support her continued healing. Furthermore, Rose has undergone training to prepare herself to work as a peer support worker. Rose’s journey as a survivor can contribute to the quality of care and support provided to individuals who struggle with mental health issues. By sharing her story and providing insight into her own recovery, she can help reduce the stigma surrounding mental health and encourage others to seek help and treatment. The success of the intervention encouraged the SRYC Institute to implement it further. Peer support intervention are currently expanding. In 2023–2024 ten adolescents struggling with suicidality will receive support from peer support workers while residing in the SRYC-facility.

## Supplementary Material

Supporting Informatiion COREQ checklist.docx

Figure 1.docx

## References

[cit0001] Alsaywid, B. S., & Abdulhaq, N. M. (2019). Guideline on writing a case report. *Urology Annals*, 11(2), 126–21. 10.4103/ua.ua_177_18 31040594 PMC6476221

[cit0002] Ambrosetti, J., Macheret, L., Folliet, A., Wullschleger, A., Amerio, A., Aguglia, A., Serafini, G., Prada, P., Kaiser, S., Bondolfi, G., Sarasin, F., & Costanza, A. (2021). Psychiatric emergency admissions during and after COVID-19 lockdown: Short-term impact and long-term implications on mental health. *BMC Psychiatry*, 21(1), 1–8. 10.1186/s12888-021-03469-8 34560856 PMC8464091

[cit0003] Amerio, A., Aguglia, A., Odone, A., Gianfredi, V., Serafini, G., Signorelli, C., & Amore, M. (2020). Covid-19 pandemic impact on mental health of vulnerable populations. *Acta Bio Medica: Atenei Parmensis*, 91(9–S), 95. 10.23750/abm.v91i9-S.10112 PMC8023095 32701924

[cit0004] Baillergeau, E., & Duyvendak, J. (2016). Experiential knowledge as a resource for coping with uncertainty: Evidence and examples from the Netherlands. *Health, Risk & Society*, 18(7–8), 407–426. 10.1080/13698575.2016.1269878

[cit0005] Braun, V., & Clarke, V. (2006). Using thematic analysis in psychology. *Qualitative Research in Psychology*, 3(2), 77–101. 10.1191/1478088706qp063oa

[cit0006] Braun, V., Clarke, V., Hayfield, N., & Terry, G. (2019). Thematic analysis. In *Handbook of research methods in health social sciences* (pp. 843–860). Springer Singapore. 10.1007/978-981-10-5251-4_103

[cit0007] Brimblecombe, N., Knapp, M., Murguia, S., Mbeah-Bankas, H., Crane, S., Harris, A., Evans-Lacko, S., Ardino, V., Iemmi, V., & King, D. (2015). The role of youth mental health services in the treatment of young people with serious mental illness: 2-year outcomes and economic implications. *Early Intervention in Psychiatry*, 11(5), 393–400. 10.1111/eip.12261 26332590

[cit0008] Buysse, W., Dickhoff, N., Faulstich, N., De Groot, M., & Hofstra, D. (2019). *Vraag en aanbod JeugdzorgPlus Factoren die van invloed zijn op de ontwikkeling in jeugdregio’s* [Supply and demand in secure residential youth Care: Factors influencing development in youth regions]. https://www.dsp-groep.nl/projecten/vraag-en-aanbod-jeugdzorgplus/

[cit0009] Byrne, L., Happell, B., & Reid-Searl, K. (2016). Lived experience practitioners and the medical model: World’s colliding? *Journal of Mental Health*, 25(3), 217–223. 10.3109/09638237.2015.1101428 26652034

[cit0010] Central Bureau of Statistics. (2020). *Overledenen; belangrijke doodsoorzaken* [Deceased individuals; significant causes of death]. Retrieved June 3, 2022 from https://opendata.cbs.nl/#/CBS/nl/dataset/80202ned/table?ts=1595575707035

[cit0011] Cha, C. B., Franz, P. J., Guzmán, E. M., Glenn, C. R., Kleiman, E. M., & Nock, M. K. (2018). Annual research review: Suicide among youth - epidemiology, (potential) etiology, and treatment. *Journal of Child Psychology and Psychiatry*, 59(4), 460–482. 10.1111/jcpp.12831 29090457 PMC5867204

[cit0012] Charmaz, K. (2014). *Constructing grounded theory* (2 ed.) Sage.

[cit0013] Chi, M. T., Long, A., Jeang, S. R., Ku, Y. C., Lu, T., & Sun, F. K. (2014). Healing and recovering after a suicide attempt: A grounded theory study. *Journal of Clinical Nursing*, 23(11–12), 1751–1759. 10.1111/jocn.12328 24251862

[cit0014] Creswell, J. W. (2018). *Qualitative inquiry & research design: Choosing among five approaches*. SAGE.

[cit0015] Davidson, L., Bellamy, C., Guy, K., & Miller, R. (2012). Peer support among persons with severe mental illnesses: A review of evidence and experience. *World Psychiatry: Official Journal of the World Psychiatric Association (WPA)*, 11(2), 123–128. 10.1016/j.wpsyc.2012.05.009 22654945 PMC3363389

[cit0016] Dazzi, T., Gribble, R., Wessely, S., & Fear, N. T. (2014). Does asking about suicide and related behaviours induce suicidal ideation? What is the evidence? *Psychological Medicine*, 44(16), 3361–3363. 10.1017/S0033291714001299 24998511

[cit0017] de Beer, C. R. M., Nooteboom, L. A., van Domburgh, L., de Vreugd, M., Schoones, J. W., & Vermeiren, R. R. J. M. (2022). A systematic review exploring youth peer support for young people with mental health problems. *European Child & Adolescent Psychiatry*, 33(8), 2471–2484. 10.1007/s00787-022-02120-5 36495354 PMC11272732

[cit0018] Deci, E. L., & Ryan, R. M. (1985). Intrinsic motivation and self-determination in human behavior. *Plenum*. 10.1007/978-1-4899-2271-7

[cit0019] de Swart, J. J. W., van den Broek, H., Stams, G. J. J. M., Asscher, J. J., van der Laan, P. H., Holsbrink-Engels, G. A., & van der Helm, G. H. P. (2012). The effectiveness of institutional youth care over the past three decades: A meta-analysis. *Children & Youth Services Review*, 34(9), 1818–1824. 10.1016/j.childyouth.2012.05.015

[cit0020] Dresen, C., Domburgh, L., Harder, A., Knorth, E., Kranenburg, M., Nijhof, K., & Vermaes, I. (2017). *Jeugdzorg met een plus: Wat we wel en nog niet weten over de meest intensieve vorm van jeugdhulp* [Youth care with a plus: What we do and don’t yet know about the most intensive form of youth assistance]. Maklu.

[cit0021] Duppong Hurley, K., Wheaton, R. L., Mason, W. A., Schnoes, C. J., & Epstein, M. H. (2014). Exploring suicide risk history among youth in residential care. *Residential Treatment for Children & Youth*, 31(4), 316–327. 10.1080/0886571X.2014.958377

[cit0022] Fisher, W. A. (1994). Restraint and seclusion: A review of the literature. *The American Journal of Psychiatry*, 151(11), 1584–1591. 10.1176/ajp.151.11.1584 7943445

[cit0023] Flyvbjerg, B. (2006). Five misunderstandings about case-study research. *Qualitative Inquiry*, 12(2), 219–245. 10.1177/1077800405284363

[cit0024] Gillard, S., Edwards, C., Gibson, S. L., Owen, K., & Wright, C. A. (2013). Introducing peer worker roles into UK mental health service teams: A qualitative analysis of the organisational benefits and challenges. *BMC Health Services Research*, 13(1), 188–188. 10.1186/1472-6963-13-188 23705767 PMC3673834

[cit0025] Glaser, B. G. (2002). Conceptualization: On theory and theorizing using grounded theory. *International Journal of Qualitative Methods*, 1(2), 23–38. 10.1177/160940690200100203

[cit0026] Glaser, B. G., & Strauss, A. L. (2008). *The discovery of grounded theory; strategies for qualitative research*. (3rd, ed.) Aldine Pub. Co.

[cit0027] Gopalan, G., Lee, S. J., Harris, R., Acri, M. C., & Munson, M. R. (2017a). Utilization of peers in services for youth with emotional and behavioral challenges: A scoping review. *Journal of Adolescence*, 55(1), 88–115. 10.1016/j.adolescence.2016.12.011 28068538

[cit0028] Gutterswijk, R. V., Kuiper, C. H. Z., Harder, A. T., Bocanegra, B. R., van der Horst, F. C. P., & Prinzie, P. (2023). Associations between secure residential care and positive behavioral change in adolescent boys and girls. *Residential Treatment for Children & Youth*, 40(2), 173–196. 10.1080/0886571X.2022.2100561

[cit0029] Harder, A. T., & Knorth, E. J. (2015). Uncovering what is inside the black box’ of effective therapeutic residential youth care. In J. K. Whittaker, J. F. del Valle, & L. Holmes (Eds.), *Therapeutic residential care with children and youth: Developing evidence-based international practice* (pp. 217–228). Jessica Kingsley Publishers.

[cit0030] Haugom, W. E., Ruud, T., & Hynnekleiv, T. (2019). Ethical challenges of seclusion in psychiatric inpatient wards: A qualitative study of the experiences of Norwegian mental health professionals. *BMC Health Services Research*, 19(1), 879. 10.1186/s12913-019-4727-4 31752958 PMC6873436

[cit0031] Heyvaert, M., Moeyaert, M., Verkempynck, P., van den Noortgate, W., Vervloet, M., Ugille, M., & Onghena, P. (2017). Testing the intervention effect in single-case experiments: A monte carlo simulation study. *The Journal of Experimental Education*, 85(2), 175–196. 10.1080/00220973.2015.1123667

[cit0032] Hill, R. M., Castellanos, D., & Pettit, J. W. (2011). Suicide-related behaviors and anxiety in children and adolescents: A review. *Clinical Psychology Review*, 31(7), 1133–1144. 10.1016/j.cpr.2011.07.008 21851804

[cit0033] Ho, K. H. M., Chiang, V. C. L., & Leung, D. (2017). Hermeneutic phenomenological analysis: The ‘possibility’ beyond ‘actuality’ in thematic analysis. *Journal of Advanced Nursing*, 73(7), 1757–1766. 10.1111/jan.13255 28103404

[cit0034] Joiner, T. E. (2005). *Why people die by suicide*. Harvard University Press.

[cit0035] Jones, P. J., Mair, P., Kuppens, S., & Weisz, J. R. (2019). An upper limit to youth psychotherapy benefit? A meta-analytic copula approach to psychotherapy outcomes. *Clinical Psychological Science*, 7(6), 1434–1449. 10.1177/2167702619858424

[cit0036] Kaijadoe, S. P. T., Klip, H., de Weerd, A., van Arragon, E. A., Nijhof, K. S., Popma, A., Scholte, R. H. J. (2023). How do group workers respond to suicidal behavior? Experiences and perceptions of suicidal female adolescents residing in secure residential youth care in the Netherlands. *PLOS ONE*, 18(3), e0283744. 10.1371/journal.pone.0283744 36996082 PMC10062624

[cit0037] Kaijadoe, S., van Arragon, E., Derksen, C., Dierick, S., de Weerd, A., Wiersma, S. (2021). *Een stil gevecht. Suïcide en suïcidaal gedrag: Wat doet dat met jou en mij?* ZonMW. https://www.zonmw.nl/fileadmin/zonmw/documenten/Jeugd/JeugdzorgPlus/eindverslag_Stil_gevecht.pdf

[cit0038] Kazdin, A. E. (1978). Methodological and interpretive problems of single-case experimental designs. *Journal of Consulting & Clinical Psychology*, 46(4), 629–642. 10.1037/0022-006X.46.4.629 670512

[cit0039] Kazdin, A. E. (2019). Single-case experimental designs. Evaluating interventions in research and clinical practice. *Behaviour Research and Therapy*, 117, 3–17. 10.1016/j.brat.2018.11.015 30527785

[cit0040] LeBel, J. L., Huckshorn, K. A., & Caldwell, B. (2010). Restraint use in residential programs: Why are best practices ignored? *Child Welfare*, 89(2), 169. 10.3928/02793695-20140915-01 20857886

[cit0041] Leemeijer, A., & Noordegraaf, M. (2020). Health professionals and peer support workers in mental health settings. In *Support workers and the health professions in international perspective: The invisible providers of health care* (pp. 143–160). Policy Press. 10.46692/9781447352112.009

[cit0042] Leipoldt, J. D., Harder, A. T., Kayed, N. S., Grietens, H., & Rimehaug, T. (2019). Determinants and outcomes of social climate in therapeutic residential youth care: A systematic review. *Children & Youth Services Review*, 99, 429–440. 10.1016/j.childyouth.2019.02.010

[cit0043] Lenkens, M., Nagelhout, G., Schenk, L., Sentse, M., Severiens, S., Engbersen, G., Dijkhoff, L., & Lenthe, F. (2020). ‘I (really) know what you mean’. Mechanisms of experiential peer support for young people with criminal behavior: A qualitative study. *Journal of Crime & Justice*, 44(5), 1–18. 10.1080/0735648X.2020.1848608

[cit0044] Mays, N., & Pope, C. (2000). Qualitative research in health care. Assessing quality in qualitative research. *BMJ*, 320(7226), 50–52. 10.1136/bmj.320.7226.50 10617534 PMC1117321

[cit0045] Mead, S., & Filson, B. (2017). Mutuality and shared power as an alternative to coercion and force. *Mental Health and Social Inclusion*, 21(3), 144–152. 10.1108/MHSI-03-2017-0011

[cit0046] Mead, S., Hilton, D., & Curtis, L. (2001). Peer support: A theoretical perspective. *Psychiatric Rehabilitation Journal*, 25(2), 134–141. 10.1037/h0095032 11769979

[cit0047] Ministerie van Volksgezondheid Welzijn en Sport. (2019). *De best passende zorg voor kwetsbare jongeren* [The most appropriate care for vulnerable young people]. Ministerie van VWS. Retrieved June 3, 2022, from https://www.rijksoverheid.nl/documenten/rapporten/2019/03/25/de-best-passende-zorg-voorkwetsbare-jongeren

[cit0048] Mulfinger, N., Müller, S., Böge, I., Sakar, V., Corrigan, P. W., Evans-Lacko, S., Nehf, L., Djamali, J., Samarelli, A., Kempter, M., Ruckes, C., Libal, G., Oexle, N., Noterdaeme, M., & Rüsch, N. (2018). Honest, open, proud for adolescents with mental illness: Pilot randomized controlled trial. *Journal of Child Psychology and Psychiatry*, 59(6), 684–691. 10.1111/jcpp.12853 29205343

[cit0049] Mulvale, G., Wilson, F., Jones, S., Green, J., Johansen, K. J., Arnold, I., & Kates, N. (2019). Integrating mental health peer support in clinical settings: Lessons from Canada and Norway. *Healthc Manage Forum*, 32(2), 68–72. 10.1177/0840470418812495 30744437

[cit0050] Niederkrotenthaler, T., Till, B., Kirchner, S., Sinyor, M., Braun, M., Pirkis, J., Tran, U. S., Voracek, M., Arendt, F., Ftanou, M., Kovacs, R., King, K., Schlichthorst, M., Stack, S., & Spittal, M. J. (2022). Effects of media stories of hope and recovery on suicidal ideation and help-seeking attitudes and intentions: Systematic review and meta-analysis. *The Lancet Public Health*, 7(2), e156–e168. 10.1016/s2468-2667(21)00274-7 35122759

[cit0051] Nock, M. K., Borges, G., Bromet, E. J., Cha, C. B., Kessler, R. C., & Lee, S. (2008). Suicide and suicidal behavior. *Epidemiologic Reviews*, 30(1), 133–154. 10.1093/epirev/mxn002 18653727 PMC2576496

[cit0052] Nolbeck, K., Wijk, H., Lindahl, G., & Olausson, S. (2020a). “If you don’t behave, you’re in real shit, you don’t get outside the doors”—a phenomenological hermeneutic study of adolescents’ lived experiences of the socio-spatial environment of involuntary institutional care. *International Journal of Qualitative Studies on Health and Well-Being*, 15(1), 1726559. 10.1080/17482631.2020.1726559 32049605 PMC7034456

[cit0053] O’Connor, R. C., & Kirtley, O. J. (2018). The integrated motivational–volitional model of suicidal behaviour. *Phil Trans R Soc B*, 373(1754), 20170268. 10.1098/rstb.2017.0268 30012735 PMC6053985

[cit0054] O’Connor, R. C., & Nock, M. K. (2014). The psychology of suicidal behaviour. *Lancet Psychiatry*, 1(1), 73–85. 10.1016/S2215-0366(14)70222-6 26360404

[cit0055] Royal College of Psychiatrists. (2010). *Helping those who self-harm*. Royal College of Psychiatrists.

[cit0056] Saldaña, J. (2016). *The coding manual for qualitative researchers* (3rd ed.). Sage.

[cit0057] Salvatore, T. (2010). Peer specialists can prevent suicides. *Behavioral Healthcare*, 30(9), 31–32. https://www.hmpgloballearningnetwork.com/site/behavioral/article/peer-specialists-can-prevent-suicides 21077545

[cit0058] Smith, J. A., Flower, P., & Larkin, M. (2009). Interpretative phenomenological analysis: Theory, method and research. *Qualitative Research in Psychology*, 6(4), 346–347. 10.1080/14780880903340091

[cit0059] Strijbosch, E. L. L., Helm, G. H. P. V. D., Brandenburg, M. E. T. V., Mecking, M., Wissink, I. B., & Stams, G. J. J. M. (2014). Children in residential care: Development and validation of a group climate instrument. *Research on Social Work Practice*, 24(4), 462–469. 10.1177/1049731513510045

[cit0060] Thomas, S. P. (2011). Editorial: Preventing suicide by using consumer peer specialists. *Issues in Mental Health Nursing*, 32(12), 725–725. 10.3109/01612840.2011.630255 22077744

[cit0061] Tong, A., Sainsbury, P., & Craig, J. (2007). Consolidated criteria for reporting qualitative research (COREQ): A 32-item checklist for interviews and focus groups. *International Journal for Quality in Health Care*, 19(6), 349–357. 10.1093/intqhc/mzm042 17872937

[cit0062] Trachtenberg, M., Parsonage, M., Shepherd, G., & Boardman, J. (2013). *Peer support in mental health: Is it good value for money?* https://www.researchgate.net/publication/308387803

[cit0063] Tucker, R. P., & Wingate, L. R. (2014). Basic need satisfaction and suicidal ideation: A self-determination perspective on interpersonal suicide risk and suicidal thinking. *Archives of Suicide Research*, 18(3), 282–294. 10.1080/13811118.2013.824839 24810541

[cit0064] van Dorp, M., Nijhof, K. S., Mulder, E. A., & Popma, A. (2021). Defining seclusion: A qualitative multiphase study based on the perspectives of youth and professionals in secure residential youth care in the Netherlands. *Residential Treatment for Children & Youth*, 38(4), 1–20. 10.1080/0886571X.2021.1879710

[cit0065] van Orden, K. A., Witte, T. K., Cukrowicz, K. C., Braithwaite, S. R., Selby, E. A., & Joiner, T. E., Jr. (2010). The interpersonal theory of suicide. *Psychological Review*, 117(2), 575–600. 10.1037/a0018697 20438238 PMC3130348

[cit0066] Vermaes, I. P. R., Konijn, C., Jambroes, T., & Nijhof, K. S. (2014). Static and dynamic characteristics of youth in secured residential care: A systematic review. *Orthopedagogiek: Onderzoek En Praktijk*, 53(6), 278–292. https://www.researchgate.net/publication/262563244_Statische_en_dynamische_kenmerken_van_jeugdigen_in_JeugdzorgPlus_Een_systematische_review_Static_and_dynamic_characteristics_of_youth_in_secured_residential_care_A_systematic_review

[cit0067] Watson, E. (2019). The mechanisms underpinning peer support: A literature review. *Journal of Mental Health*, 28(6), 677–688. 10.1080/09638237.2017.1417559 29260930

[cit0068] Whittaker, J. K., Del Valle, J. F., & Holmes, L. (2015). *Therapeutic residential care for children and youth: Developing evidence-based international practice*. Jessica Kingsley Publishers.

[cit0069] Whittaker, J. K., Holmes, L., Del Valle, J. F., Ainsworth, F., Andreassen, T., Anglin, J., Bellonci, C., Berridge, D., Bravo, A., Canali, C., Courtney, M., Currey, L., Daly, D., Gilligan, R., Grietens, H., Harder, A., Holden, M., James, S., Kendrick, A., & Zeira, A. (2016). Therapeutic residential care for children and youth: A consensus statement of the international work group on therapeutic residential care. *Residential Treatment for Children & Youth*, 33(2), 89–106. 10.7334/psicothema2016.172 28693697

[cit0070] World Health Organization. (2021). *Suicide worldwide in 2019: Global health estimates*. Retrieved June 1, 2022, from https://www.who.int/publications/i/item/9789240026643

[cit0071] Zuidersma, M., Riese, H., Snippe, E., Booij, S. H., Wichers, M., & Bos, E. H. (2020). Single-subject research in psychiatry: Facts and fictions. *Frontiers in Psychiatry*, 11, 539777. 10.3389/fpsyt.2020.539777 33281636 PMC7691231

