# Supplementary figures and images for: “Surviving against the odds. The impact of peer support workers on a chronically suicidal adolescent in secure residential youth care: a single case report from the Netherlands”
Source: Int J Qual Stud Health Well-being. 2024 Oct 8;19(1):2409514. doi: 10.1080/17482631.2024.2409514 (PMC11463016; doi:10.1080/17482631.2024.2409514)

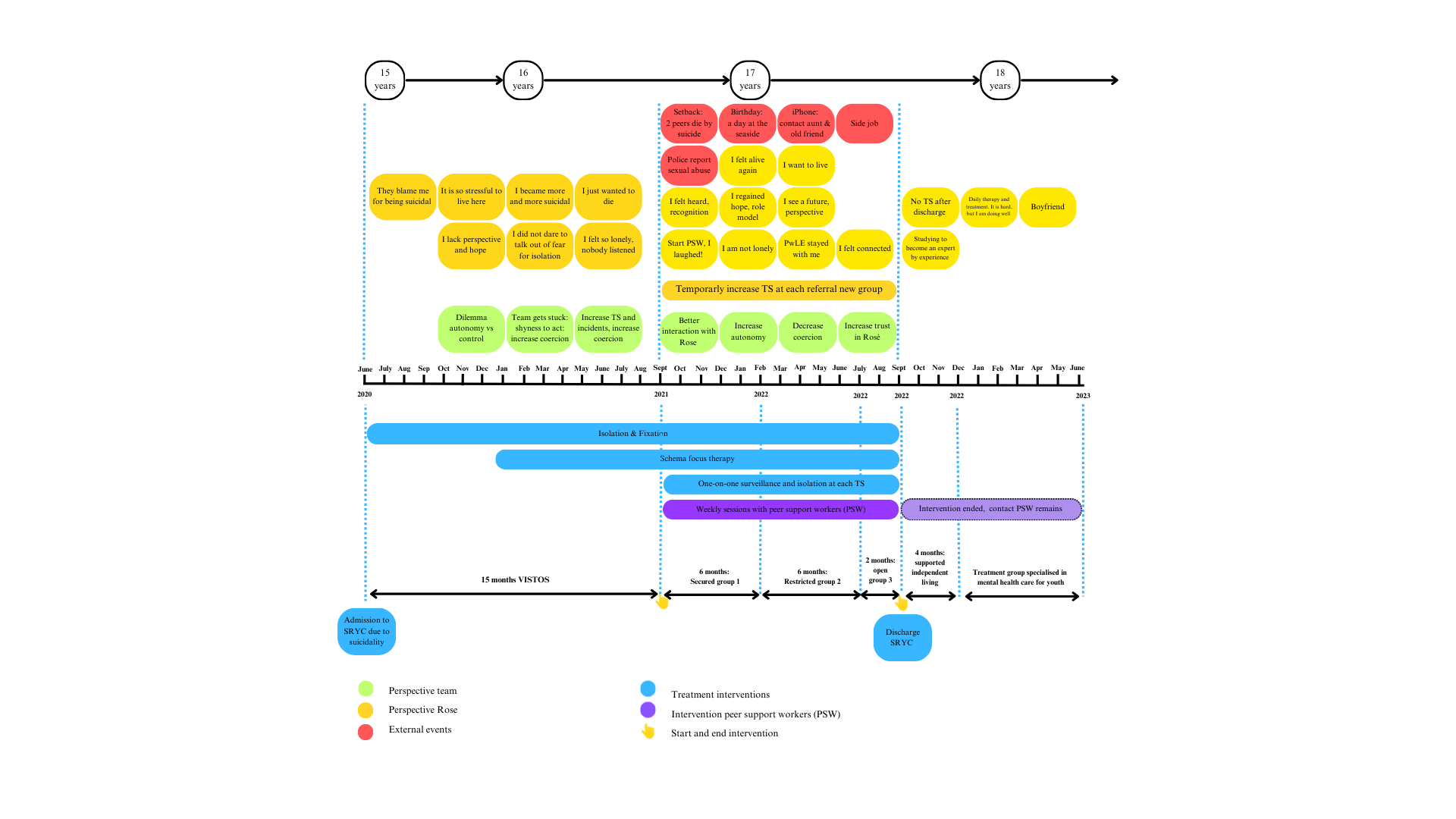


**Figure 1. Rose’s patient journey**

Supplement: Figure 1.docx [file ZQHW_A_2409514_SM9252.docx]
